# Modified pedicle screw-rod versus anterior subcutaneous internal pelvic fixation for unstable anterior pelvic ring fracture: a retrospective study and finite element analysis

**DOI:** 10.1186/s13018-021-02618-9

**Published:** 2021-07-27

**Authors:** Zhi-Hong Pan, Fan-Cheng Chen, Jun-Ming Huang, Cheng-Yi Sun, Sheng-Long Ding

**Affiliations:** 1 Zhoujiadu Community Health Service Center, Pudong New Area, Shanghai, 200126 People’s Republic of China; 2 Department of Foot Ankle Surgery Beijing Tongren Hospital, Capital Medical University, No. 1, Dongjiaomin Lane, DongCheng District, Beijing, 100730 People’s Republic of China; 3 Department of Orthopaedic Surgery, Zhongshan Hospital, Fudan University, Shanghai, 200032 People’s Republic of China

**Keywords:** Pelvic fracture, Anterior ring, High-energy trauma, Minimally invasive fixation

## Abstract

**Objectives:**

This study compared the stability and clinical outcomes of modified pedicle screw-rod fixation (MPSRF) and anterior subcutaneous internal pelvic fixation (INFIX) for the treatment of anterior pelvic ring fractures using the Tornetta and Matta grading system and finite element analyses (FEA).

**Methods:**

In a retrospective review of a consecutive patient series, 63 patients with Orthopaedic Trauma Association (OTA)/Arbeitsgemeinschaft für Osteosynthesefragen (AO) type B or C pelvic ring fractures were treated by MPRSF (*n* = 30) or INFIX (*n* = 33). The main outcome measures were the Majeed score, incidence of complications, and adverse outcomes, and fixation stability as evaluated by finite element analysis.

**Results:**

Sixty-three patients were included in the study, with an average age of 34.4 and 36.2 in modified group and conventional group, respectively. Two groups did not differ in terms of the injury severity score, OTA classification, cause of injury, and time to pelvic surgery. However, the MPSRF group had a rate of higher satisfactory results according to the Tornetta and Matta grading system than the conventional group (73.33% vs 63.63%) as well as a higher Majeed score (81.5 ± 10.4 vs 76.3 ± 11.2), and these differences were statistically significant at 6 months post-surgery. FEA showed that MPSRF was stiffer and more stable than INFIX and had a lower risk of implant failure.

**Conclusions:**

Both MPSRF and INFIX provide acceptable biomechanical stability for the treatment of unstable anterior pelvic ring fractures. However, MPSRF provides better fixation stability and a lower risk of implant failure, and can thus lead to better clinical outcomes. Therefore, MPSRF should be more widely applied to anterior pelvic ring fractures

**Supplementary Information:**

The online version contains supplementary material available at 10.1186/s13018-021-02618-9.

## Introduction

Anterior pelvic structures are more fragile and prone to fracture than dorsal structures as they bear higher loads [1]. Clinically, high-energy pelvic ring fractures leading to decreased functionality account for approximately 1.5–3.9% of all fractures [2]. The high rates of morbidity and mortality are of concern to orthopedists and place an economic burden on patients and society.

External fixation and open reduction internal fixation (ORIF) are the primary modalities of treatment for trauma to the extremities and pelvis, which could provide a rapid and stiff fixed strength [3]. However, these methods are associated with complications such as pin tract infection (in 2–50%), fixation loosening (in 0–20%), loss of reduction (0–30%), and restriction of daily activities, particularly in obese patients [4, 5]. Moreover, open reduction carries the potential disadvantage of extensive exposure including muscle stripping, as well as a risk of damage to neurovascular structures. Therefore, minimally invasive fixation methods are increasingly being used as an alternative to external fixation methods for the treatment of anterior pelvic ring fractures [6, 7]. The insertion of supra-acetabular pedicle screws connected via a subcutaneous contoured rod tunneled just below the belly crease (the so-called bikini area) [8], which is known as the subcutaneous anterior pelvic fixation (INFIX) technique, has the advantages of convenience, minimal invasiveness and blood loss, and relatively little discomfort for patients with anterior pelvic ring injuries [9, 10].

The evidence for its use, unfortunately, is also limited. INFIX was first designed for obese patients and has gradually promoted the application of non-obese patients with anterior pelvic ring fractures because of its minimally invasive characteristics. However, this weakened the stability of the fixation due to the lack of a part of the support effect of the fat and the rod could produce obvious micro-movements with the patient’s activities. Many aspects require improvement such as the persistence of pubic pain, soft tissue irritation, loss of reduction, and especially fixator loosening [5, 11]. The most common complication reported was lateral femoral cutaneous nerve palsy (occurred temporarily in 0–30%) and heterotopic ossification (reported in 0–25%) [5, 7]. In order to improve the fixation strength, an additional pubic ramus pedicle screw was added, which we defined modified pedicle screw-rod fixation (MPSRF). More importantly, we improved this surgical technique to reduce the compression of vascular nerves and tissue irritation.

We previously showed that MPSRF can lead to more rapid recovery from anterior pelvic ring fractures, and patients can obtain greater clinical outcome [12]. However, it is unclear how MPSRF compares to INFIX in terms of strength and stability. We hypothesized that MPSRF could provide sufficient biomechanical stability compared with INFIX. To test this hypothesis, we examined the postoperative biomechanical characteristics of the implants when patients assumed single-/dual-leg standing and sitting postures by finite element analysis (FEA), a computational method that has been used to assess and predict the outcome of surgery that could take many various factors into consideration including the bone quality, fracture pattern, bone anatomy, and fixation location upon application of physiological loads [13–15].

## Methods

### Patients and methods

**Fig. 1:**
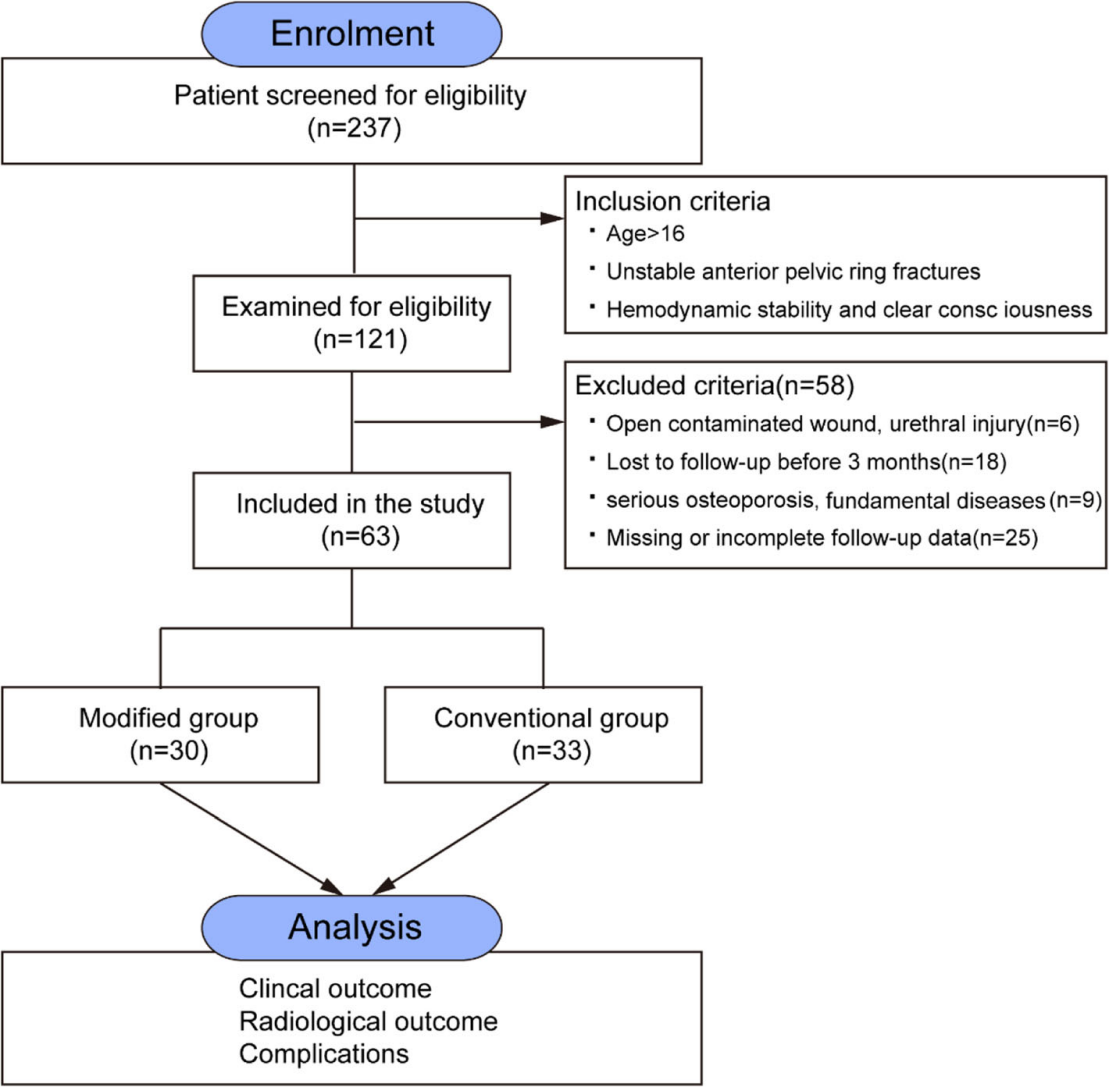
Flow diagram of patient’s exclusion. Flow diagram of patient’s exclusion. Sixty-three patients with anterior pelvic ring injuries were enrolled from January 2014 to January 2017 in our institution

This retrospective study was reviewed and approved by the Ethics Committee of Zhongshan Hospital. All procedures were performed in accordance with the Declaration of Helsinki and strictly adhered to institutional guidelines. A total of 63 patients with anterior pelvic ring injury were enrolled from January 2014 to January 2017, with a minimum follow-up of 13 months. The inclusion criteria were unstable anterior pelvic ring fracture with a stable posterior ring (either intact or recovered after fixation) diagnosed by the senior trauma surgeon, hemodynamic stability, and full consciousness. Patients with an open contaminated wound, who were lost to follow-up before 3 months, who had a pathological fracture, or who were < 16 years of age were excluded (Fig. 1).

The pelvis of each patient was examined by preoperative radiography (including anterior-posterior [AP], inlet, and outlet views) and computed tomography (CT). Imaging data were analyzed by 2 senior orthopedists according to the Arbeitsgemeinschaft für Osteosynthesefragen (AO) and Orthopaedic Trauma Association (OTA) modified tile type classification. Post-surgery clinical outcome was assessed based on Tornetta and Matta grade, Majeed score, and complications.

### Surgical technique

If required, surgical reduction of the posterior pelvic ring injury was performed prior to fixation as previously described [12]. For MPSRF, a 3- to 4-cm oblique incision was made over each anterior inferior iliac spine (AIIS). A bony tunnel from the AIIS to the posterior superior iliac spine was created with a pedicle finder. A polyaxial pedicle screw with a diameter of 7 mm and length of 60 mm was inserted into the tunnel to a depth of approximately 2 cm from the bone surface to avoid compressing vascular tissue. A subcutaneous tunnel was created from the incisions over the bilateral AIIS to the Pfannenstiel incision over the deep fascia. The curved titanium rod was inserted to connect the 3 bilateral pedicle screws via the subcutaneous tunnel. After confirming that there was sufficient space between the rod and bone by fluoroscopy, the screws were tightened with a torque screwdriver, with those at bilateral AIIS tightened before the one at the pubic tubercle. A representative case is shown in Supplementary Figure 1.

The INFIX was inserted in the same manner. Briefly, 2 polyaxial pedicle screws were placed at bilateral AIIS. A subcutaneous tunnel was created from 1 side of the AIIS to the other, and the precontoured titanium rod was connected to the 2 screws via the tunnel. Fracture reduction was performed by the same method as described above. A representative case is shown in Supplementary Figure 2.

### Postoperative management and follow-up

Functional exercises of the lower limbs and joints were initiated in bed as early as possible after the operation to prevent deep vein thrombosis; regular wound and dressing care was performed in the outpatient clinic until sutures were removed at 2 weeks postoperatively. Crutch-assisted partial weight bearing was permitted at 6 and 10 weeks for AO/OTA type B and C injuries, respectively, as long as the pain was tolerable. Full weight bearing was allowed if osseous union was confirmed by radiography. Physical therapy was prescribed for muscle strengthening and gait training. Hardware removal was performed between 12 and 14 months after the surgery.

Patients at the clinic routine follow-ups were scheduled for postoperative weeks 4 and 8 and months 6 and 12, and then were followed up by phone at postoperative in months 18 and 24. Radiographic images included a 3-view (AP, inlet, and outlet) pelvis series, and all patients were asked about pain, numbness, and motor ability at each follow-up. Physical and neurologic examinations were performed to evaluate irritation around the implant as well as the condition of the lateral femoral cutaneous nerve (LFCN) and femoral nerves.

### Radiographic and functional outcome assessment

The extent of fracture reduction was evaluated by postoperative X-ray examination and graded according to the method of Tornetta and Matta [16] as excellent (displacement ≤ 4 mm), good (5–10 mm), fair (10–20 mm), or poor (displacement > 20 mm). A grade of excellent or good was regarded as a satisfactory outcome.

Clinical outcome measures included the operation time, hospital stay, intraoperative blood loss, and postoperative complications. The Majeed rating system was used to assess functional outcome at 6 months, the time of implant removal (10–14 months), and the last follow-up; the variables were pain (30 points), standing (36 points), sitting (10 points), sexual intercourse (4 points), and work (20 points). Aggregate scores were classified as excellent (>85), good (70–84), fair (55–69), or poor (< 55) [17].

### FEA

To define the solid geometry of the pelvis, we constructed a model of the pelvis of a healthy 32-year-old male (height, 175 cm; weight, 73 kg) based on CT images. The material properties of the model were obtained from previous studies (Table 3) [1]. A 2-cm gap was created at the right superior and inferior rami to simulate injury using Geomagic Studio software (3D Systems Inc., Rock Hill, SC, USA). 3D models of the rod and screws were constructed using Creo version 3.0 software (Parametric Technology Corp, Needham, MA, USA). The materials for the different models and implants were assumed to be elastic, isotropic, and linear. A value of 0.33 was set as Poisson’s ratio (y) for both sacrum cortical and ilium cortical bone (Table 3).

Models of the intact pelvis and injured pelvis treated with the 2 types of fixation were imported into Workbench version 17.0 software (ANSYS Inc., Canonsburg, PA, USA) to analyze equivalent von Mises (VM) stress and displacement. We performed convergent analysis to balance the accuracy and efficiency of the finite element simulation by adjusting element size. Interaction surfaces including sacrum, sacroiliac cartilage ilium, pubic rami, and bone implant were fully constrained and a vertical load of 600 N was applied as a distribution over an area on the superior surface of sacrum to simulate upper body weight. The unilateral (left/right) acetabulum, bilateral acetabulum, and bilateral ischium were fully constrained to simulate single-leg (left/right) and dual-leg standing and sitting postures, respectively (Supplementary Figure 3).

### Statistical analysis

Data were analyzed using SPSS version 20.0 software (SPSS Inc., Chicago, IL, USA). Data satisfying the conditions of normality are presented as means ± standard deviation; non-normal data are presented as medians and quartiles. Differences in categorical variables (e.g., postoperative complications) were assessed with the chi-squared test or Fisher’s exact test, whereas differences in continuous variables were evaluated with Student’s *t* test when the assumption of normality was valid. A *P* value < 0.05 was considered significant.

## Results

### Clinical outcome

A consecutive series of 63 patients with type B and type C fractures were enrolled. The causes of injury were traffic accidents (*n* = 28), falls (*n* = 21), and other (*n* = 14). The average age and the sex ratio did not differ between the two groups. The mean injury severity score of patients who underwent INFIX was 25 points (range, 19–29 points); that of patients who underwent MPSRF was 22 points (range, 19–29 points). The 2 groups were similar with respect to OTA classification and causes of injury as well as time to pelvic surgery. However, the conventional group had longer operation time and greater blood loss than the MPSRF group. There was also a statistically significant difference between the two groups in terms of hospital stay (Table 1).

**Table 1:** The demographics of two groups (ISS, injury severity score)

Parameter	Modified group (*n* = 30)	Conventional group (*n* = 33)	*P* value
Age(years)	34.4 ± 17.5	36.2 ± 16.9	0.856^a^
Gender (male/female)	16/14	20/13	0.560^b1^
ISS	22(19, 29)	25(21, 32)	0.283^a^
AO/OTA/modified tile type			0.136^b2^
B1/B2/B3	4/15/6	7/17/3	
C1/C2/C3	2/3/0	4/2/0	0.846^b1^
Injury mechanism			
Traffic injury	13	15	
Falling injury	11	10	
Other	6	8	
Time to surgery (days)	5(0, 14)	6(0,16)	0.782^d^
Operation time (min)	77.3 ± 11.2	52.6 ± 12.9	0.015^a^
Intraoperative blood loss (ml)	103.6 ± 10.2	87.7 ± 9.3	0.036^a^
Hospital stays (days)	11(7, 18)	14(8, 21)	0.028^a^

^a^Two-sample *t* test

^b1^Pearson chi-squared test

^b2^Cochran-Mantel-Haenszel test

^c^Fisher’s exact test

^d^Log rank test

The median follow-up was 20 months (range, 13–27 months) in the conventional group and 22 months (range, 17–30) in the modified group. All patients’ fractures healed without incident after the operation. Regarding the Tornetta and Matta grade, the modified group had rate of satisfactory results ([“excellent” + “good”]/total number of patients) compared to the conventional group (73.33% vs 63.63%), but the difference was not statistically significant (*P* = 0.409). The modified group had a higher Majeed score than the conventional group at 6 months postoperatively (*P* < 0.001) and at the time of implant removal (*P* = 0.012). However, there was no difference in the scores of the two groups at 22 months (Table 2).

**Table 2:** Postoperative functional outcome grading and complications

	Modified group (*n* = 30)	Conventional group (*n* = 33)	*P* value
Tornetta and Matta grading			0.037^b2^
Excellent	12	6	0.409^b1^
Good	13	15	
Fair	4	9	
Poor	1	3	
Satisfactory rate	22/30, (73.33%)	21/33, (63.63%)	
The Majeed score			
Postoperatively 6 months	81.5 ± 10.4	76.3 ± 11.2	< 0.001^a^
The time of implant removal	84.8 ± 8.63	80.7 ± 7.37	0.012^a^
The last follow-up (22 months)	88.4 ± 7.12	87.6 ± 6.45	0.065^a^
Postoperative complications (*n*)	12	14	0.307^c^
LFCN irritation (*n*)	10	8	
Femoral nerve palsy (*n*)	1	0	
Tardive impingement pain (*n*)	0	1	
Infection	1	2	
Implant loosening	0	3	

^a^Student’s *t* test

^b1^Pearson chi-squared test

^b2^Cochran-Mantel-Haenszel test

^c^Fisher’s exact test

### Complications

LFCN irritation was observed in 10 patients (33.3%) in the modified group and 8 patients (24.2%) in the conventional group. The symptoms mainly manifested as anterolateral skin numbness of the affected thigh. In most cases, this was alleviated by physical therapy at 6 months postoperatively, and nearly two-thirds of the 26 patients recovered after implant removal. There was 1 case of unilateral femoral nerve palsy immediately after the operation in the modified group, and 1 patient complained of tardive impingement pain without motor dysfunction at 3 months’ post-surgery in the conventional group. Additionally, 3 patients who underwent INFIX experienced implant loosening (Table 2).

### Biomechanical characteristics of intact model

We constructed, meshed, and solved models of an intact pelvis and an injured pelvis treated with 2 types of fixation in single- (right/left) and dual-leg standing and sitting postures. Displacement distribution and VM stress of the intact pelvis were bilaterally symmetrical and cantered on the sacrum in the dual-leg standing and sitting postures. The maximum stress was 28, 30, 21, and 18 MPa on the right face of the sacrum in the single-leg (left/right) and dual-leg standing and sitting postures, respectively (Supplementary Figure 4B).

### Evaluation of fixation stability in the injury model

**Fig. 2:**
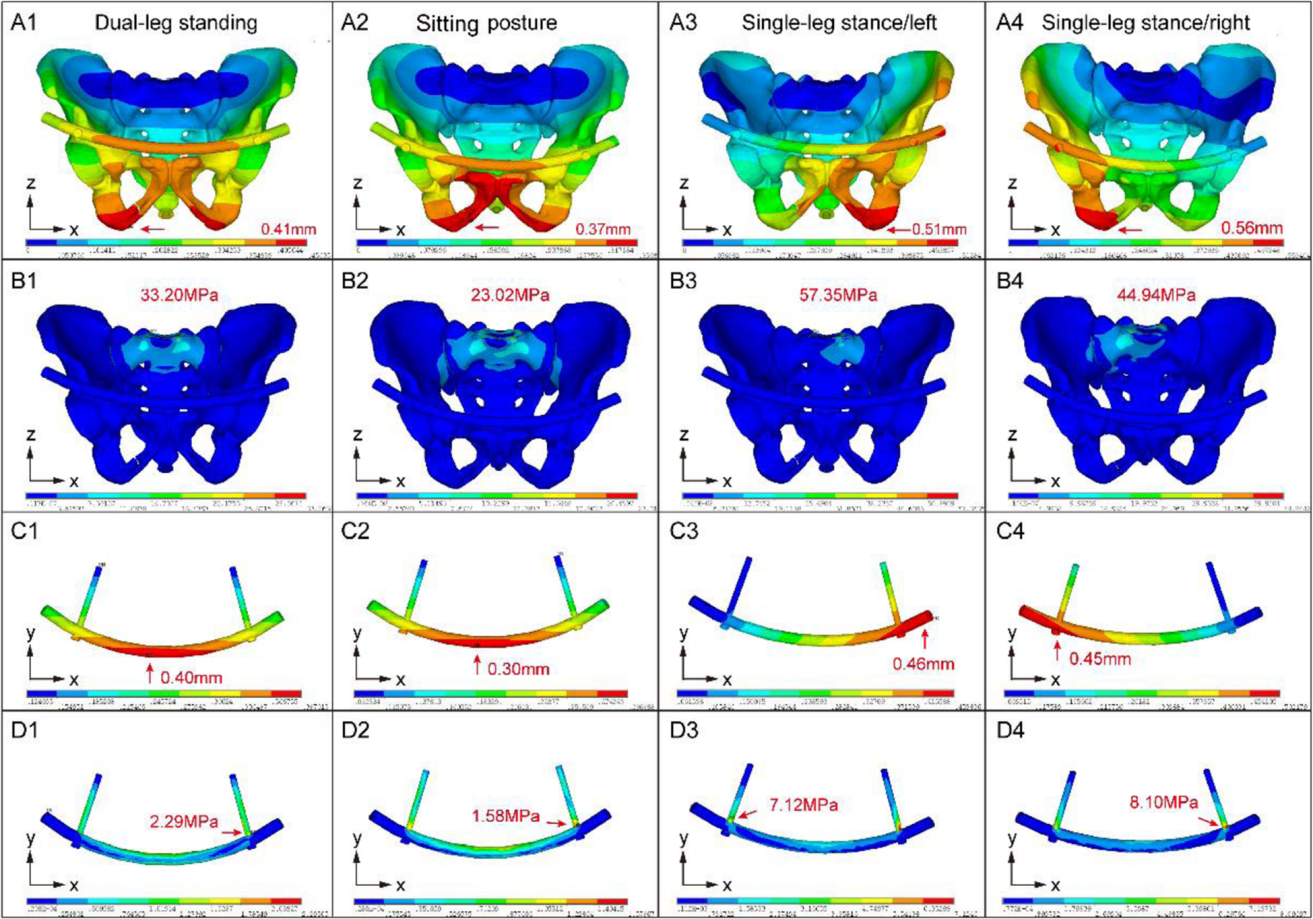
FE injured model using INFIX under four posture, including the dual-leg standing, sitting posture and single-leg stance (left/right). **A** The displacement of the injured pelvis with INFIX. **B** The von Mises stresses applied to the injured pelvis with INFIX. **C** The displacement of the implant device. **D** The von Mises stresses applied to the implant device

**Fig. 3:**
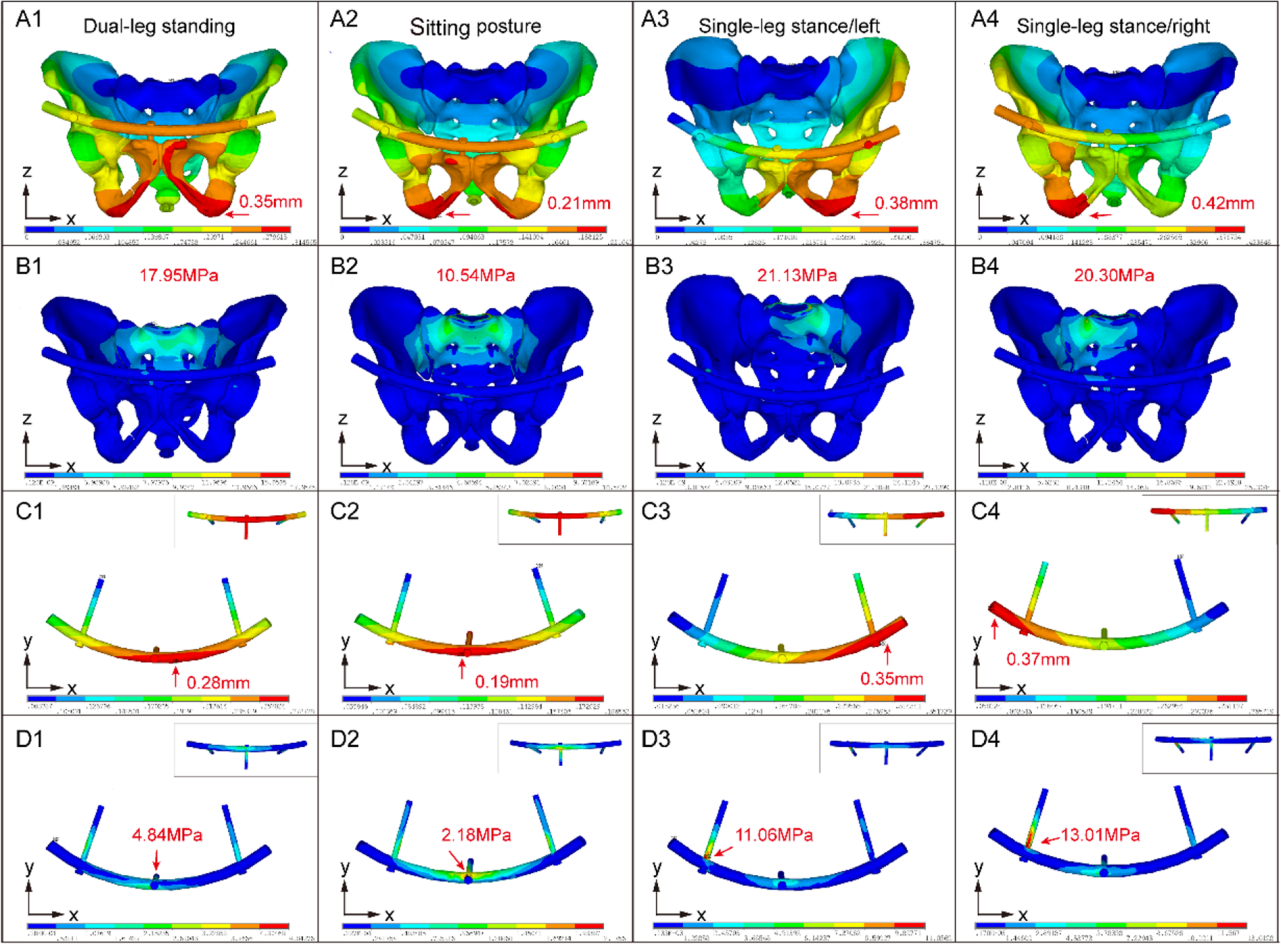
FE injured model with MPSRF under four posture, including the dual-leg standing, sitting posture and single-leg stance (left/right). **A** The displacement of the injured pelvis with MPSRF. **B** The von Mises stresses applied to the injured pelvis with MPSRF. **C** The displacement of the implant device. **D** The von Mises stresses applied to the implant device

A lower maximum displacement of the pelvis in the model indicates greater stability, in general or due to fixation. Among the finite element models in the 4 postures, the MPSRF model had a lower maximum pelvic displacement than that the INFIX model, especially in the sitting posture (0.37 vs 0.21 mm) and the single-leg standing posture (left, 0.51 vs 0.38 mm; right, 0.56 vs 0.42 mm), indicating that MPSRF provides greater stability (Supplementary Figure 4). Moreover, the maximum pelvic displacement of the MPSRF model was comparable to that of the intact pelvis model. The maximum VM stress was slightly higher in the INFIX model on the face of the sacrum than that of the MPSRF and intact models, with a maximum VM stress in the single-leg (left) posture of 57.3 MPa vs 21 and 28 MPa, respectively (Figs. 2 and 3), which is consistent with the observed trend in maximum pelvic displacement. Thus, the injured pelvis showed greater stiffness when treated by MPSRF than INFIX.

### Risk of implant failure and micromotion in the injury model

A lower maximum implant displacement indicates a lower risk of implant failure and micromotion and higher fixation stability. For the finite element models in the 4 postures, the injury model treated by MPSRF had 30% and 36% lower maximum displacement compared to the INFIX-treated model in the standing posture (0.40 mm vs 0.28 mm) and sitting posture (0.30 mm vs 0.19 mm), respectively, indicating a lower risk of implant failure and micromotion (Supplementary Figure 4).

**Table 3:** Material properties used in the finite element models

Material	Elastic modulus (MPa)	Poisson ratio (μ)
Sacrum cortical bone	6200	0.33
Ilium cortical bone	17,000	0.33
Sacrum cancellous bone	1400	0.30
Ilium cancellous bone	130	0.20
Screws/rod	110,000	0.30
Cartilage*	60	0.30

*The cartilage at the pubic symphysis

A lower maximum vM stress in the implant represents a lower risk of pelvic breakage. Maximum implant stress was slightly higher in the injury model treated by MPSRF compared to INFIX in all 4 postures (Supplementary Figure 4). However, maximum M stress occurred at the central region of the third pedicle screw of the pubis in the model treated by MPSRF in the standing and sitting postures (Figs. 2 and 3D), implying a lower risk of micromotion. In the single-leg stance/right posture, the maximum VM stress was on the side opposite to the implant in the INFIX model but on the same side in the MPSRF model (Figs. 2 and 3D). Taken together, the FEA results indicate that anterior pelvic ring fractures treated by MPRSF are stiffer, have greater fixation stability, and lower risk of implant failure than those treated by INFIX.

## Discussion

Unstable pelvic ring injuries typically result from high-energy trauma and are considered by orthopedic surgeons to be one of the most clinically challenging lesions. Open reduction and internal fixation are the standard operative treatment for hemodynamically stable patients with anterior pelvic ring injuries. Nevertheless, major issue concerning this method are poor patient tolerance leading to frequent complaints when patients present decubitus, pin tract infections, and inability to sit. To address these drawbacks, minimally invasive fixation of pelvic fractures as an adjunct to posterior fixation is becoming more common and can effectively improve anterior pelvic stability and restore pelvic function; additionally, it is associated with minimal trauma and fewer complications [18]. INFIX is a technique in which pedicle screws are placed on the supra-acetabular corridor with a crossrod in the bikini area [19]. INFIX has recently emerged as an alternative to traditional surgical methods with the advantages of few soft tissue complications, improved biomechanical construction, and no disruption of sitting posture or screwdriver function. MPSRF combines the advantages of INFIX and pelvic bridging. The safety and efficacy of minimally invasive fixation of pelvic fractures have been demonstrated by several anatomical and clinical studies, although fixation stability remains a concern [20, 21]. Additionally, few studies have investigated whether these two fixation techniques lead to sufficiently strong implants and adequate biomechanical reduction.

The FEA results of the present study show that the displacement distribution and vM stress were similar in the MPSRF and INFIX models compared to the intact model, indicating that both fixation techniques can effectively treat anterior pelvic ring fractures. Song et al. reported that the maximum von Mises stress appeared at the rod-screw and screw-bone interfaces in floating public symphysis by finite element analysis, which is basically consistent with our result analysis [22]. However, the maximum displacements of the pelvis and implant were lower in the modified group than in the conventional group in the single- (left/right) and dual-leg standing and sitting postures, indicating greater stability. On the other hand, the MPSRF model showed a slightly higher maximum implant stress than the INFIX model in all four positions, which may be attributable to part of the stress being concentrated on the third screw and obstructing implant micromotion.

Although the modified group had a longer operation time and greater intraoperative blood loss, there were no significant differences in hospital stay and postoperative complications between the 2 groups. Thus MPSRF only resulted in expansion of the local skin incision without causing permanent neurovascular injury and did not increase the risk of postoperative complications. However, radiographic (Tornetta and Matta grade) and functional (Majeed score) outcomes were better in the modified group than in the conventional group, especially at 6 months after surgery. Thus, while both fixation methods can restore anterior pelvic ring stability and pelvic function, MPSRF promotes the latter at an earlier time point following the operation.

LFCN irritation is the most common postoperative iatrogenic complication [23]. In a multicenter review, 30% (21/91) of patients had LFCN irritation although in most cases it was self-limiting and improved once the implant was removed [5]. Similarly, in our investigation, the rate of LFCN injury was 33.3% (10/30) in the modified group and 24.2% (8/33) in the conventional group. A case series of LFCN irritation suggested that screws that are too deeply or insufficiently embedded in the bone and inadequate prebending of the rod can lead to irritation of the LFCN and sartorius muscle. To prevent this, a rod-to-bone distance of 20–25 mm (30–40 mm for obese patients) but < 40 mm is recommended [24]. We first locked the screws bilaterally at AIIS so that the pull-out strength of the screw was mainly concentrated in the supra-acetabular region, where bone density is high. The screw at the pubic tubercle—where the bone is relatively sparse—was then locked, thus providing auxiliary support. Furthermore, additional screws should not be placed too close to the lateral pubis to avoid damaging the spermatic cord or round ligament. For unilateral pubic rami fractures, the screw was fixed into the fracture side if the fracture line was far away from the pubic symphysis; otherwise, it was inserted on the uninjured side. For bilateral pubic rami fractures, the screw was fixed into the side with less injury.

**Fig. 4:**
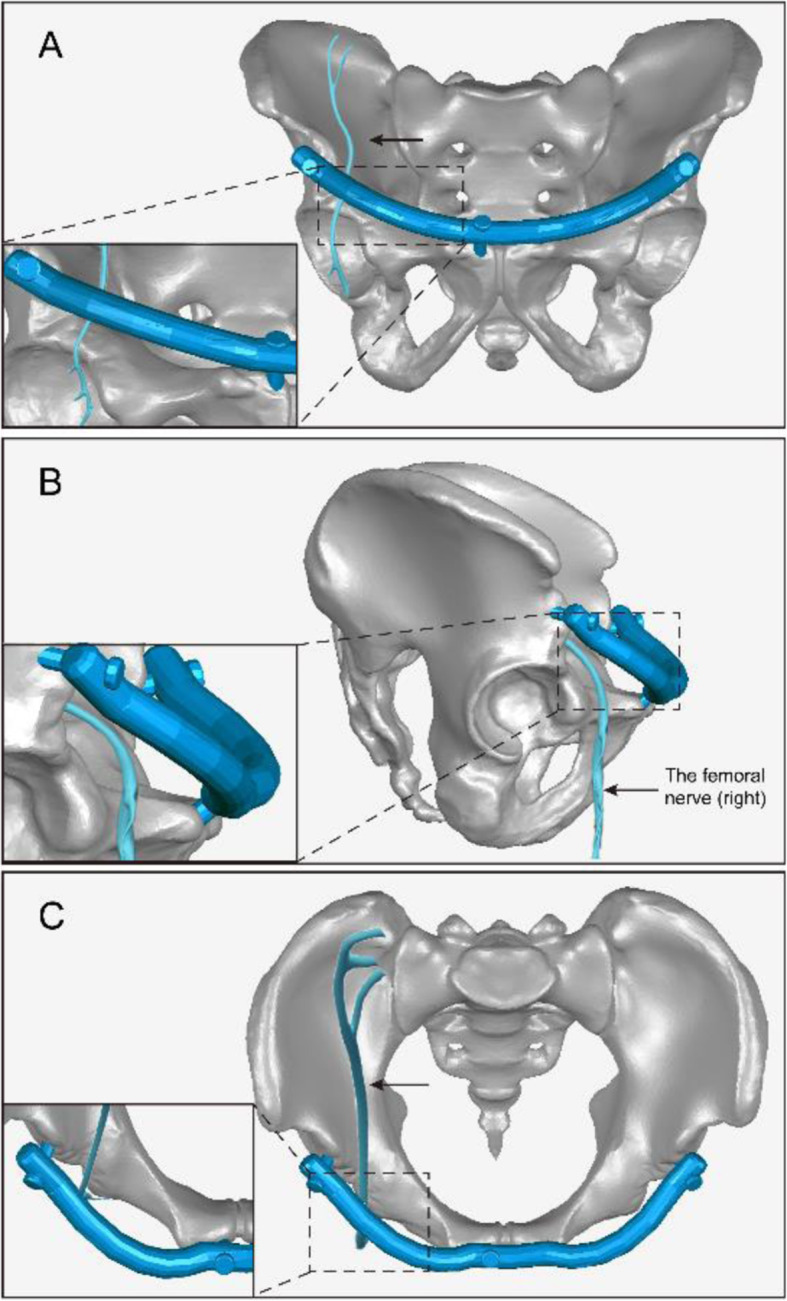
The diagram of the improvement applied to the MPSRF. The diagram of the improvement applied to the MPSRF, bending the connecting rod outward in the horizontal direction

Recent studies using the INFIX or MPSRF technique have reported potentially devastating complications, especially femoral nerve palsy. There was one such case in the modified group, but symptoms gradually disappeared once emergency screw adjustment was performed, and there was no permanent nerve damage after implant removal. In a case series of iatrogenic femoral nerve palsy, it was suggested that femoral nerve compression occurs as a result of impingement of the implant on the psoas sheath; meanwhile, delayed palsy may be caused by engorgement of the psoas with blood and/or a change in pressure [25]. The authors noted that this could be avoided by placing the interconnecting rod in such a way that it does not limit the space for the psoas and femoral nerve. Our solution for reducing neurovascular compression was to bend the connecting rod outward in the horizontal direction (Fig. 4). Although follow-up is on-going, the patient’s neurovascular compression symptoms have been significantly alleviated.

Despite the positive clinical outcomes and FEA results, our study has several limitations. First, this was a single-center retrospective study with a relatively small sample size; more cases should be examined in a multicenter investigation, with long-term functional assessment. Second, the characteristics of the implant materials are not consistently reported; as such, our results depend on input parameters. Additionally, the FEA model was used to assess one subject, with homogeneous material properties, and one fracture pattern, and ignored the effects of ligaments and muscles on the stability of the pelvis and implant; meanwhile, the material properties for bone and implant were obtained from the literature rather than measurements and the reconstructed grafts were assumed to be homogeneous isotropic linear elastic. Finally, the angle of applied force was constant although variations in the angle may have influenced the vM stress and displacement, as reported in a previous study [26].

## Conclusion

In conclusion, our study demonstrates that both MPSRF and INFIX provide adequate biomechanical stability as minimally invasive procedures for the treatment of unstable anterior pelvic ring fractures. However, MPSRF provided greater fixation stability than INFIX, which was associated with a lower risk of implant failure according to the FEA results. Combined with the superior clinical and radiological outcomes associated with external fixation, MPSRF is the preferred option for the treatment of unstable anterior pelvic ring fractures.

## Supplementary Information

**Additional file 1: Supplementary Figure 1**. **A** 48-year-old female patient with anterior pelvic ring fracture. **A** and **B**. Preoperative X-ray film and 3D CT image showed bilateral displaced pubic ramus fracture. **C**. Postoperative pelvic AP view, the X-ray film showed satisfactory reduction with the modified three-screw fixation. **D**. the Pelvic AP view of X-ray film showed bone union at six months’ follow-up, postoperatively. **Supplementary Figure 2**. **A** 45-year-old female patient with anterior pelvic fracture. **A** and **B** Preoperative X-ray film and 3D CT image showed left pubic ramus fracture. **C**. Postoperative pelvic AP view, the X-ray film showed satisfactory reduction with the conventional two-screw fixation. **D**. Pelvic AP view of X-ray film showed bone union at seven months’ follow-up, postoperatively. **Supplementary Figure 3**. FE intact model under four posture. **A**: geometries for model with different loading stress. **B**: The displacement of the intact pelvis. **C**: The Von Mises stresses distribution applied to the intact pelvis. **Supplementary Figure 4**. Comparison of fixation stability in intact model and injured model with INFIX/MPSRF under four postures including the dual-leg standing, sitting posture and single-leg stance(left/right). **A**: the maximum displacement of pelvis and implant devices. **B**: maximum Von Mises stresses of pelvis and implant devices.

## Data Availability

The datasets used and/or analyzed during the current study are available from the corresponding author on reasonable request.

## Abbreviations

AIIS: Anterior inferior iliac spine
AO: Arbeitsgemeinschaft für Osteosynthesefragen
AP: Anterior-posterior
CT: Computed tomography
FEA: Finite element analysis
INFIX: Anterior subcutaneous internal pelvic fixation
LFCN: Lateral femoral cutaneous nerve
MPSRF: Modified pedicle screw-rod fixation
OTA: Orthopaedic Trauma Association
vM: von Mises
