# Supplementary material for: Modified pedicle screw-rod versus anterior subcutaneous internal pelvic fixation for unstable anterior pelvic ring fracture: a retrospective study and finite element analysis
Source: J Orthop Surg Res. 2021 Jul 27;16:467. doi: 10.1186/s13018-021-02618-9 (PMC8314600; doi:10.1186/s13018-021-02618-9)
Supplement: Supplementary file 1 — Additional file 1: Supplementary Figure 1. A 48-year-old female patient with anterior pelvic ring fracture. A and B. Preoperative X-ray film and 3D CT image showed bilateral displaced pubic ramus fracture. C. Postoperative pelvic AP view, the X-ray film showed satisfactory reduction with the modified three-screw fixation. D. the Pelvic AP view of X-ray film showed bone union at six months’ follow-up, postoperatively. Supplementary Figure 2. A 45-year-old female patient with anterior pelvic fracture. A and B Preoperative X-ray film and 3D CT image showed left pubic ramus fracture. C. Postoperative pelvic AP view, the X-ray film showed satisfactory reduction with the conventional two-screw fixation. D. Pelvic AP view of X-ray film showed bone union at seven months’ follow-up, postoperatively. Supplementary Figure 3. FE intact model under four posture. A: geometries for model with different loading stress. B: The displacement of the intact pelvis. C: The Von Mises stresses distribution applied to the intact pelvis. Supplementary Figure 4. Comparison of fixation stability in intact model and injured model with INFIX/MPSRF under four postures including the dual-leg standing, sitting posture and single-leg stance(left/right). A: the maximum displacement of pelvis and implant devices. B: maximum Von Mises stresses of pelvis and implant devices. [file 13018_2021_2618_MOESM1_ESM.docx]

**Supplement information**


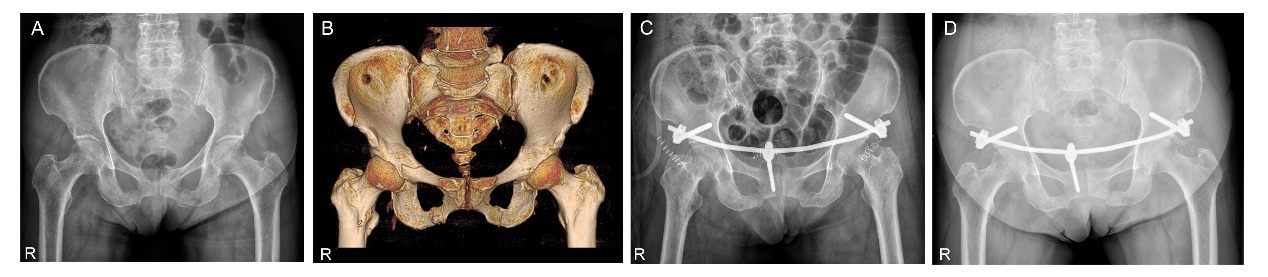


Supplement figure 1. A 48-year-old female patient with anterior pelvic ring fracture. A and B. Preoperative X-ray film and 3D CT image showed bilateral displaced pubic ramus fracture. C. Postoperative pelvic AP view, the X-ray film showed satisfactory reduction with the modified three-screw fixation. D. the Pelvic AP view of X-ray film showed bone union at six months’ follow-up, postoperatively.


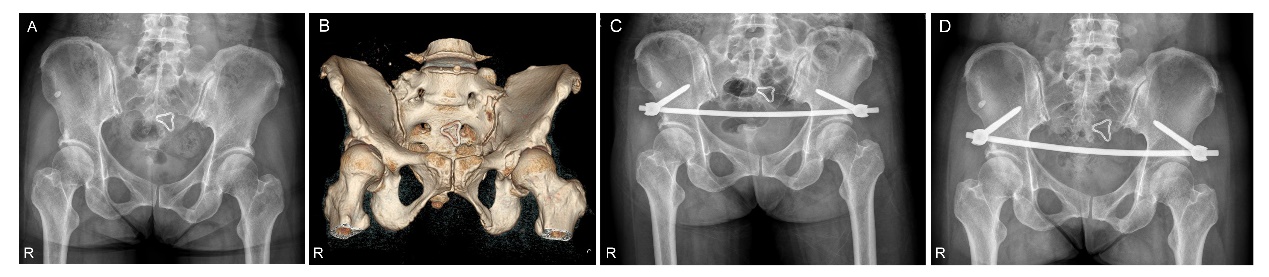


Supplement figure 2. A 45-year-old female patient with anterior pelvic fracture. A and B Preoperative X-ray film and 3D CT image showed left pubic ramus fracture. C. Postoperative pelvic AP view, the X-ray film showed satisfactory reduction with the conventional two-screw fixation. D. Pelvic AP view of X-ray film showed bone union at seven months’ follow-up, postoperatively.


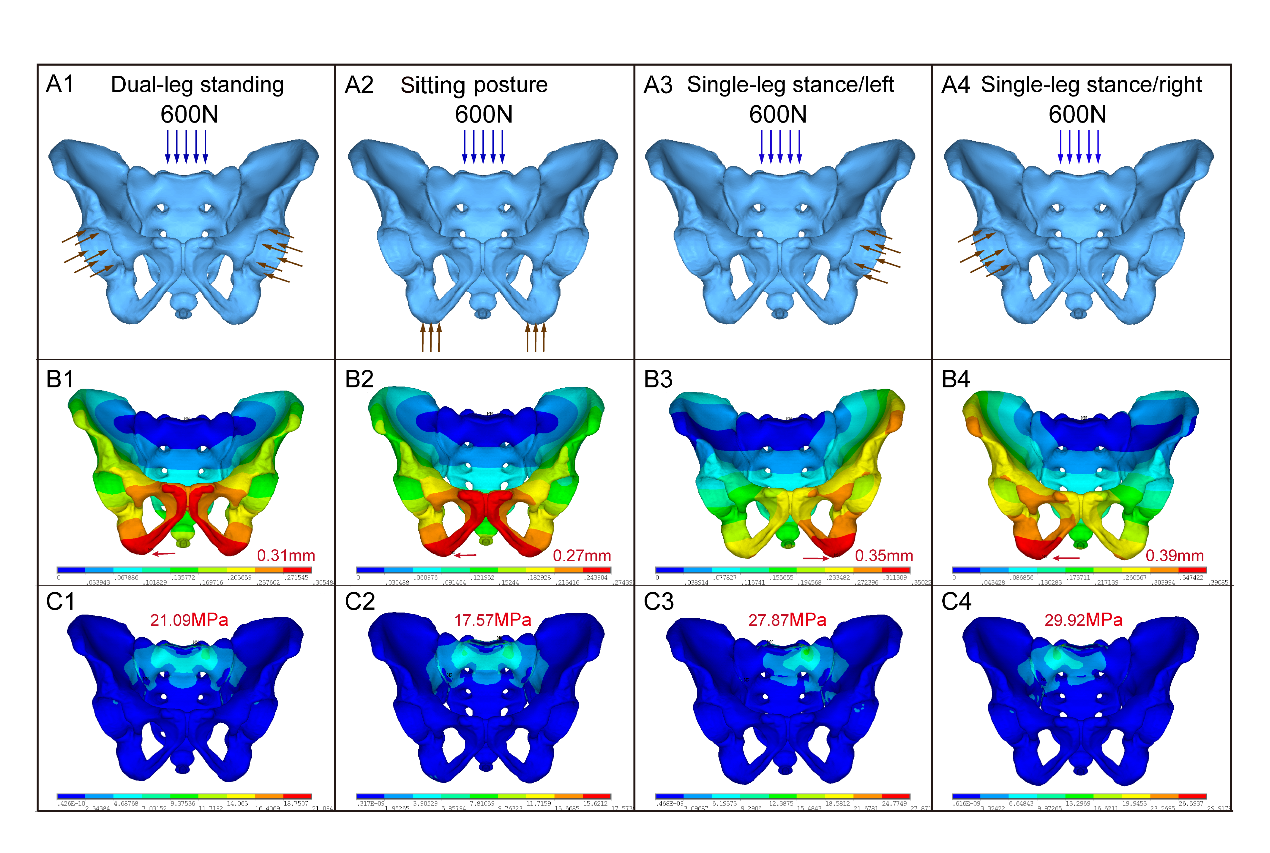


Supplement Figure 3. FE intact model under four posture. A: geometries for model with different loading stress. B: The displacement of the intact pelvis. C: The Von Mises stresses distribution applied to the intact pelvis.


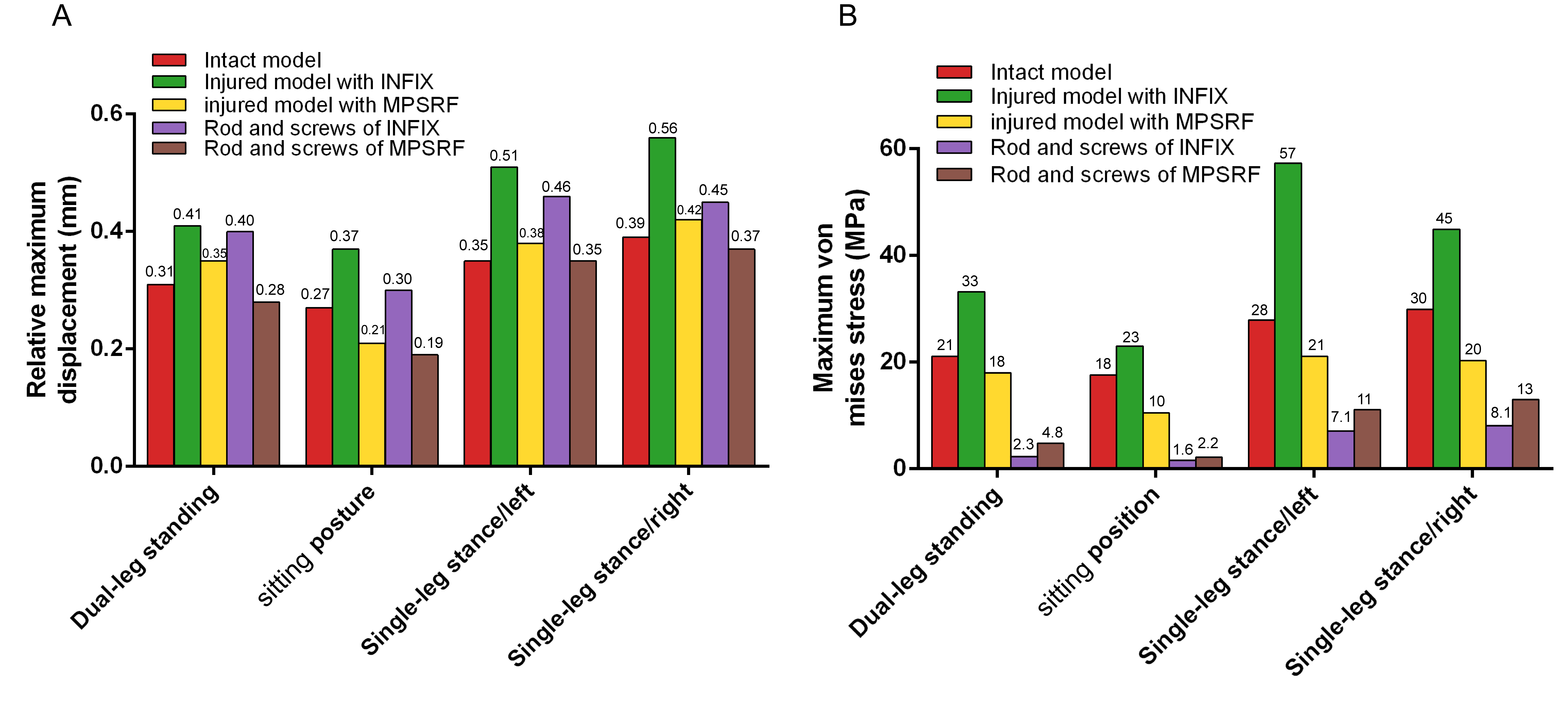


Supplement figure 4. Comparison of fixation stability in intact model and injured model with INFIX/MPSRF under four postures. including the dual-leg standing, sitting posture and single-leg stance(left/right). A: the maximum displacement of pelvis and implant devices. B: maximum Von Mises stresses of pelvis and implant devices.
